# Operational Research Improves Compliance with Treatment Guidelines for Empirical Management of Urinary Tract Infection: A Before-and-After Study from a Primary Health Facility in Ghana

**DOI:** 10.3390/tropicalmed10090259

**Published:** 2025-09-11

**Authors:** Elizabeth Boateng, Helena Owusu, Pruthu Thekkur, George Kwesi Hedidor, Oksana Corquaye, Mercy N. A. Opare-Addo, Florence Amah Nkansah, Priscilla Vandyck-Sey, Daniel Ankrah, Charles Nii Kwadee Ofei-Palm

**Affiliations:** 1 Pharmacy Department, Korle Bu Teaching Hospital, Accra P.O. Box KB77, Ghana; h.owusu@kbth.gov.gh (H.O.); oksana_corquaye@yahoo.com (O.C.); a.nkansah@kbth.gov.gh (F.A.N.); d.ankrah@kbth.gov.gh (D.A.); c.ofei-palm@kbth.gov.gh (C.N.K.O.-P.); 2 Korle Bu Polyclinic/Family Medicine Department, Accra P.O. Box KB77, Ghana; p.vandyck@kbth.gov.gh; 3 Centre for Operational Research, International Union Against Tuberculosis and Lung Disease (The Union), 75001 Paris, France; pruthu.tk@theunion.org; 4 World Health Organization Country Office, Accra P.O. Box MB142, Ghana; hedidorg@who.int; 5 Department of Pharmacy Practice, Kwame Nkrumah University of Science and Technology, Kumasi P.O. Box 509, Ghana; mnaopare-addo.pharm@knust.edu.gh

**Keywords:** antimicrobial resistance, antimicrobial stewardship, clinical audit, audit–feedback mechanism, quality of care, evidence-based medicine, operational research, SORT IT

## Abstract

Compliance with standard treatment guidelines (STGs) for the empirical management of uncomplicated urinary tract infections (UTIs) ensures the rational use of antibiotics and mitigates antimicrobial resistance. Operational research (OR) at Korle Bu Polyclinic in Ghana (2022) showed suboptimal STG compliance (prescription of recommended antibiotics in the correct dose, route, frequency, and duration). Some of the recommendations from the OR, including training of medical residents, implementation of an antimicrobial stewardship team, and an audit–feedback system, were implemented. This before-and-after study compared the changes in empirical prescribing practices for patients diagnosed with uncomplicated UTIs before (October 2019–October 2021) and after (January 2023–December 2024) the OR. Of the 3717 and 3457 UTI patients in the before and after cohorts, 83% and 86% received empirical antibiotics, respectively. Among those who received empirical antibiotics, STG compliance increased from 60% to 66% (*p*-value < 0.001). However, STG compliance remained significantly lower among males (18%) compared to females (85%) in the after cohort, as 80% of males were prescribed antibiotics for a shorter period than the recommended 10–14 days. Moving forward, the training and audit–feedback system should emphasize longer antibiotic durations for males with UTI. Given its positive impact, the OR’s approach warrants replication for other disease conditions.

## 1. Introduction

Urinary tract infections (UTIs) are the second most common infectious disease after upper respiratory tract infections [1]. It is estimated that about 50% of women and 12% of men develop UTIs at least once in their lifetime [2]. Globally, an estimated 150 million people develop UTIs each year, resulting in more than six billion dollars of direct healthcare expenditures [2]. In 2019, nearly 240,000 patients died from complications related to UTI worldwide [3]. Though about 80% of UTIs are uncomplicated, without structural or functional abnormalities in the urinary tract, if not treated promptly, they can progress to life-threatening consequences such as complicated UTIs, pyelonephritis, and sepsis [4].

Global guidelines recommend empirical antibiotics for the management of suspected uncomplicated UTIs as they help in the early resolution of symptoms and prevent complications compared to placebo or non-steroidal anti-inflammatory drugs [5,6]. Empirical antibiotics form the mainstay of management of uncomplicated UTIs in lower-and-middle-income countries (LMIC) with poor access to definitive diagnosis with culture and drug susceptibility testing (CDST) [7,8]. Thus, countries are advised to develop standard treatment guidelines (STGs) with specific guidance on empirical antibiotics based on the antibiogram of uropathogens, costs, efficacy, and availability of the antibiotics in the country [7,8,9].

Globally, most countries have STGs for uncomplicated UTIs [1,10]. Previous studies from the United States, Sweden, Netherlands, China, and Lebanon have reported poor compliance with STGs on empirical antibiotics for management of uncomplicated UTIs [11,12,13,14,15,16,17,18]. Such poor compliance could lead to suboptimal management of UTIs and irrational use of antibiotics, contributing to antimicrobial resistance [19].

In Ghana, the STGs (7th Edition, 2017) recommend empirical oral ciprofloxacin or cefuroxime for 5–7 days for females and 10–14 days for males for the management of uncomplicated UTI [20]. Until 2022, there were no studies on compliance with the STGs in the management of uncomplicated UTI in primary health facilities of Ghana. Through the Structured Operational Research Training Initiative (SORT IT) [21,22], Owusu et al. [23] conducted operational research to assess compliance with the STGs in prescribing empirical antibiotics for adults diagnosed with uncomplicated UTI at the Korle Bu Polyclinic/Family Medicine Department (KBPFMD, hereafter referred to as KBP) between 2019 and 2021.

Owusu et al. [23] found that of 3717 patients with uncomplicated UTI, 83% were prescribed empirical antibiotics, of which only 60% were prescribed the drug, dose, frequency and duration in line with the STGs. This low compliance was mainly due to the prescription of empirical antibiotics for a duration not in line with the STGs, especially among male patients who required antibiotics for 10–14 days. To improve compliance with STGs in KBP, the investigators recommended instituting an audit–feedback system, exploring the reasons for poor compliance, and promoting similar efforts for assessing compliance with STGs in managing other common conditions [23]. The investigators also recommended that the Ministry of Health (MoH) revise the STGs by introducing Access group antibiotics for the empirical management of uncomplicated UTIs and consider reducing the treatment duration for male patients. However, revisions to the STGs are ongoing and will be implemented soon.

The principal investigator (PI) actively disseminated the operational research findings and recommendations to the decision-makers of the KBP using knowledge management aids developed through SORT IT [24]. Following active dissemination, some of the recommendations were partially or completely implemented at the KBP (as detailed in Section 2). However, there was a need for a formal assessment to explore whether the implementation of recommendations from the operational research had improved compliance with the STGs. Thus, we conducted an audit of empirical antibiotic use among adult patients (≥18 years) diagnosed with uncomplicated UTIs at the KBP during January 2023 to December 2024 (after cohort) to compare the proportion that was prescribed empirical antibiotics, the pattern of empirical antibiotics used across World Health Organization’s AWaRe categories, and the proportion of prescribed empirical antibiotics in line with the STGs with those found among those diagnosed during October 2019 to October 2021 (before cohort).

## 2. Materials and Methods

### 2.1. Study Design

This was a before (October 2019–October 2021) and after (January 2023–December 2024) comparison of cross-sectional assessments conducted using routinely collected electronic data of patients with uncomplicated UTI diagnosed at the KBP.

### 2.2. Study Setting

#### 2.2.1. General Setting

Ghana, an LMIC, is located in West Africa, with a population of 34 million according to the World Population Prospects 2023 [25]. The country is divided into 16 administrative regions, with Greater Accra being the capital [25]. Ghana’s healthcare system operates within a three-tier structure: primary, secondary, and tertiary [26]. The healthcare system is mostly managed by the government through the National Health Insurance Scheme (NHIS). The NHIS supports outpatient services, inpatient services, laboratory investigations, and medicines for about 95% of the common diseases in Ghana [27,28].

#### 2.2.2. Specific Setting

The KBP is the primary care arm of the Korle Bu Teaching Hospital, the largest teaching hospital in Ghana. The KBP is a 42-bed facility in the capital city of Accra and provides healthcare services to the local community and the staff of Korle Bu Teaching Hospital. The KBP offers various services, including 24-hour emergency care, eye care, radiology, pharmacy, laboratory, child immunization, and minor surgeries. About 200 outpatients visit KBP daily. Approximately 90% of outpatients are enrolled under the NHIS, significantly reducing out-of-pocket expenditures for healthcare services [28].

##### Management of Uncomplicated UTIs at the KBP

The diagnosis and treatment of uncomplicated UTI are supposed to align with the STGs of Ghana [20]. UTIs are suspected based on the patient’s presenting complaints and/or routine examination of mid-stream urine. Though CDST is advised in the STGs for the management of uncomplicated UTI, it is hardly ever used by the treating physicians, mainly because of cost and the non-availability of tests at the in-house laboratory. Thus, patients with suspected uncomplicated UTIs are usually treated with empirical antibiotics and antipyretic drugs on an ambulatory basis. According to the STGs, patients with suspected uncomplicated UTIs may be prescribed empirical oral antibiotics, either ciprofloxacin or cefuroxime. For adults, when empirical antibiotics are prescribed, these have to be ciprofloxacin 500 mg every 12 h for 7 days in females and 10–14 days in males or cefuroxime 250–500 mg every 12 h for 5–7 days in females and 10–14 days in males [20]. Both ciprofloxacin and cefuroxime, recommended in the STGs, are part of the essential drug list of the KBTH and are mostly available throughout the year. The pharmacy at the KBP also has several other oral antibiotics available. Individuals with complicated UTIs are usually admitted and treated with intravenous antibiotics.

##### Electronic Medical Records

The KBP has been using an electronic medical record (EMR) system since 2019. The socio-demographic, clinical, laboratory and pharmacy details of all the outpatients are captured in the EMRs. The socio-demographic details of outpatients are captured at the registration desk, and a unique ID is generated. The clinician enters the clinical details into the EMRs, including the provisional diagnosis according to the International Classification of Diseases-10 (ICD-10) [29]. Then, the clinician makes an electronic prescription detailing the drug (generic name), dose, frequency, and duration for each medication prescribed. The electronic prescription is accessed at the pharmacy to dispense the prescribed medicines to the patients. If some prescribed medications are unavailable, an electronic prescription is printed and issued to the patient for purchase elsewhere. In addition, all the laboratory investigation results are available as scanned copies against the patient visit.

#### 2.2.3. Dissemination of Findings of the Operational Research Study

As mentioned earlier, Owusu et al. [23] conducted a study to assess compliance with the STGs in prescribing empirical antibiotics for uncomplicated UTIs. The PI disseminated the study findings and the recommendations to the decision-makers and key stakeholders of the KBP. The published article and the dissemination materials developed during module 4 (module on communicating research findings) of SORT IT were used for dissemination. The dissemination details are reported in Table 1.

#### 2.2.4. Recommendations Made and Actions Taken

Following the effective dissemination of information, some of the recommendations made by Owusu et al. [23] were translated into actions. In Table 2, we have mapped the recommendations made and actions taken, as reported by the PI of the previous study.

### 2.3. Study Population

All adults (aged ≥ 18 years) who were diagnosed with uncomplicated UTI (with ICD-10 code of N39.0) in the outpatient department of the KBP from January 2023 to December 2024 were included in the study. Patients with complicated UTIs, those with urethral catheters, pregnant women, those with urologic abnormalities, repeat visits within 30 days of initial treatment, and inpatients were excluded from the study. Owusu et al. [23] also included a similar study population during October 2019 to October 2021.

Assuming that compliance would increase by at least 5% (60% to 65%) in the after period, with a confidence level of 95% and a power of 80%, the minimum sample size needed for the study was calculated to be 1490 outpatients. However, we included all outpatients diagnosed with uncomplicated UTI during the study period.

### 2.4. Data Collection, Sources, and Variables

In January 2025, the principal investigator liaised with the Information Technology (IT) unit of the KBP and downloaded the registration, OPD consultation, and pharmacy prescription details of all uncomplicated UTI outpatients (ICD-10 code of N39.0) between January 2023 and December 2024 from the EMR. Pregnant women and inpatients were excluded before downloading. These data were then screened and filtered to remove all ineligible participants (repeat visits within 30 days of initial treatment). In addition, information on prescriber details (gender and rank), routine urine examination (done/not done), and co-morbidities (diabetes and/or hypertension) were extracted manually from the EMRs by two independent researchers, since these were not part of the extracted data. Similar efforts were made by Owusu et al. [23] while extracting data for the previous operational research study and the database was directly used for analysis.

#### Operational Definitions

Prescribed any empirical antibiotic: All uncomplicated UTI patients who have been prescribed at least one antibiotic in their first prescription.

Prescribed empirical antibiotic drug recommended in STGs: All uncomplicated UTI patients who have been prescribed either ciprofloxacin or cefuroxime in their first prescription.

Prescribed empirical antibiotics in line with STGs: All uncomplicated UTI patients who have been prescribed either ciprofloxacin or cefuroxime in the dose, route, frequency, and duration as per the STGs.

### 2.5. Data Analysis

The downloaded data in Microsoft Excel format were cleaned and imported into Stata (version 16.0, Copyright 1985–2019, StataCorp LLC, College Station, TX, USA) for analysis. The socio-demographic, clinical, and prescriber characteristics were categorized and summarized using frequencies and percentages. The chi-square test was used to compare the socio-demographic, clinical, and prescriber characteristics in the before (October 2019 to October 2021) and after (January 2024 to December 2024) cohorts.

Numbers and percentages were used to describe the patients with uncomplicated UTIs prescribed an empirical antibiotic, prescribed an empirical antibiotic drug recommended in the STGs, and those prescribed empirical antibiotics in line with the STGs. The chi-square test was used to compare these percentages across the before and after cohorts. Numbers and percentages were used to describe the pattern of empirical antibiotics used across the WHO AwaRe (Access, Watch, and Reserve) categories. The chi-square test was used to compare these percentages across the before and after cohorts.

Log binomial regression was used for unadjusted analysis in the after cohort to assess the association of patient and prescriber characteristics with those “prescribed empirical antibiotics in line with the STGs”. We conducted an adjusted analysis using modified Poisson regression with all the patient and prescriber characteristics included. The prevalence ratios (PRs) and adjusted prevalence ratios (aPRs) with 95% CIs were presented as the measure of association.

## 3. Results

### 3.1. Socio-Demographic and Clinical Characteristics

In total, 3457 adults diagnosed with uncomplicated UTIs in the outpatient department of the KBP between January 2023 and December 2024 were included in the reassessment conducted “after” the operational research (after cohort). The mean age of the patients was 49.5 (19.9) years; 2483 (71.8%) were females and 2718 (78.6%) were covered under the NHIS. Of the 3457 patients, 644 (18.6%) and 1327 (38.4%) had diabetes and hypertension, respectively. The prescriber was a male for 1783 (51.6%) patients and held the rank of medical officer for 1466 (42.4%) patients (Table 3).

The socio-demographic characteristics of the “before” cohort are described in the manuscript published by Owusu et al. [23]. There was no statistically significant difference in age, sex and NHIS coverage among patients diagnosed in the before (October 2019 to October 2021) and after (January 2024 to December 2024) cohorts. There was a statistically significant difference in the distribution of hypertension (33.1% vs. 38.4%), diabetes (16.7% vs. 18.6%), and routine urine examination (69.3% vs. 67.1%) among patients included in the before and after cohorts. Similarly, there was a statistically significant difference in the distribution of prescriber characteristics (Table 3).

### 3.2. Prescription of Empirical Antibiotics for UTI Patients

**Figure 1:**
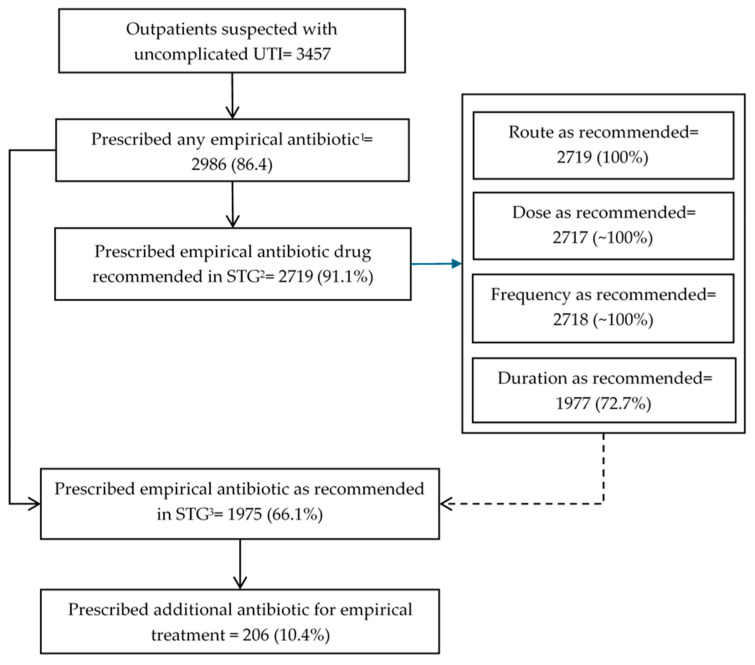
Flowchart depicting empirical antibiotic prescription for outpatients suspected of having uncomplicated UTI and guideline concordance among prescribers at Korle Bu Polyclinic/Family Medicine Department of Ghana during January 2023 to December 2024. ^1^ Prescription of antibiotic of any class by the treating physician. ^2^ Prescription of either oral ciprofloxacin or cefuroxime, as recommended in the STGs. ^3^ Prescription of either oral ciprofloxacin or cefuroxime in at the correct dose, route, frequency, and duration, as recommended in the STGs. Abbreviations: UTI = urinary tract infection; STGs = standard treatment guidelines.

In the after cohort of 3457 patients, 2986 (86.4%) received an empirical antibiotic. Among these, 2719 (91.1%) were prescribed antibiotics recommended in the STGs. Nearly all these 2719 patients received the correct dose, frequency, and route, as specified in the STGs. However, only 72.7% of patients received antibiotics for the recommended duration according to the STGs, with a notably low proportion among males (20%). Among the 2986 patients prescribed an empirical antibiotic, 1975 (66.1%) received treatment that met the STG recommendations for dose, route, frequency, and duration. Additionally, 10% of these patients were prescribed concurrent antibiotics (Figure 1). A corresponding description for the before cohort is provided in the manuscript by Owusu et al. [23].

Compared with the before cohort, the after cohort showed a statistically significant increase in the proportion of patients with uncomplicated UTI who were prescribed empirical antibiotics (83.0% to 86.4%, *p* < 0.001), the STG-recommended empirical antibiotic (88.0% to 91.1%, *p* < 0.001), and the recommended treatment duration (68.0% to 72.7%). The proportion of patients receiving empirical antibiotics fully compliant with the STGs also increased significantly (60.0% to 66.1%, *p* < 0.001), representing a 10% relative improvement (Table 4).

### 3.3. Distribution of Prescribed Empirical Antibiotics Across the WHO AWaRe Category

In the before cohort, 3378 empirical antibiotics were prescribed for 3073 patients (1.1 per patient), compared with 3491 empirical antibiotics prescribed for 2986 patients in the after cohort (1.2 per patient). The proportion of Access group antibiotics increased significantly from 11.2% in the before cohort to 16.5% in the after cohort (*p* < 0.001). Significant increases were observed in the use of Access group antibiotics such as nitrofurantoin (2.7% to 4.1%), doxycycline (2.6% to 4.5%), and metronidazole (0.2% to 2.0%). In both cohorts, cefuroxime and ciprofloxacin were the most frequently prescribed antibiotics. However, cefuroxime use declined significantly (54.2% to 42.4%), while ciprofloxacin use increased (30.7% to 35.9%) between the before and after cohorts (Table 5).

### 3.4. Patient and Prescriber Characteristics Associated with the Prescription of Empirical Antibiotics Not in Line with the STGs

In the after cohort, on adjusted analysis, males (aPR-5.4, 95% CI: 4.9–6.1), compared to females, had a significantly higher risk of being prescribed empirical antibiotics not as recommended in STGs. None of the other patient characteristics (age, NHIS status, presence of comorbidities, and routine urine examination) or prescriber characteristics (gender and rank) were independently associated with being prescribed empirical antibiotics not as recommended in the STGs (Table 6).

## 4. Discussion

This before-and-after study showed an increase in compliance with the STGs in prescribing empirical antibiotics for uncomplicated urinary tract infections in a primary health facility in Ghana, following the implementation of recommendations of an operational research by Owusu et al. [23]. There were some key programmatic changes that we noticed in the after cohort (January 2023–December 2024) compared to the before cohort (October 2019–October 2021) after the operational research was conducted. First, there was a 10% relative increase (from 60% to 66%) in compliance with the STGs. Second, there was a significant increase in the prescription of empirical antibiotics. Third, there was an increase in the prescription of the WHO Access category antibiotics. Finally, males still had a five times higher risk of being prescribed empirical antibiotics not in line with the STGs, primarily because antibiotics were not prescribed for a longer duration (10–14 days).

Antimicrobial resistance (AMR) contributes to an estimated five million annual deaths globally, with the highest burden of death in the Western Sub-Saharan African region [30]. Thus, there is an urgent need to develop and implement effective strategies for promoting the rational use of antimicrobials to tackle the emergence of AMR [31]. This study from Ghana has shown how insights from an audit through operational research can increase the rational use of antibiotics by improving compliance with the STGs for uncomplicated UTI, which is one of the top ten causes of OPD visits in the country [32]. Hence, the study justifies the WHO’s call to implement audit–feedback systems in the healthcare facilities of LMICs, as well as the utility of operational research in applying it [33].

This study has some strengths. First, we used the same inclusion criteria, data source, operational definitions, STGs, and statistical package to assess compliance with the STGs in both before and after cohorts, improving the internal validity of the comparison. Secondly, the substantial sample size provided the statistical power to accurately assess adherence to the STGs and compare it between the before and after cohorts. Thirdly, the EMR data used in the study were complete, without any missing values. Finally, we adhered to the STROBE (Strengthening the Reporting of Observational Studies in Epidemiology) guidelines for reporting study findings [34].

This study has some limitations. First, not having a control group limited our ability to entirely attribute the improvement in compliance with the implementation of the recommendations of the before study. However, as the time difference between the two assessments was short and no other changes were put in place to enhance service delivery in the facility, the improvement can likely be attributed to the implementation of the recommendations. Second, the prescriber and patient characteristics (hypertension and routine urine examination) were not comparable between the before and after cohorts. As the prescriber and patient characteristics were not independently associated with compliance with the STGs in both the before and after cohorts, the effect of the differences in prescriber characteristics on the compliance comparison may be negligible. Third, due to the unavailability of data, we audited the prescriptions by the treating physicians, rather than the actual drug dispensation process by the pharmacist, to describe compliance with the STGs. However, physicians only prescribe the drugs available in the facility, and the pharmacist dispenses all prescribed medications. Fourth, although the previous study recommended future audits to assess the uptake of CDST, antibiograms, and antibiotic switching when required, difficulties in integrating laboratory data prevented such exploration. Finally, given that the study was conducted in a single primary care facility functioning under a teaching hospital, its findings may not be readily generalizable to other primary care settings in the country.

Despite these limitations, the study findings have some important implications for antimicrobial stewardship in primary care settings.

First, the significant increase in empirical antibiotic prescriptions for uncomplicated UTIs in the after cohort might be due to the introduction of the “STG orientation with clinical audit” in the medical residents’ training programme, as recommended by Owusu et al. [23]. Although the STGs recommend that empirical antibiotics may be prescribed while awaiting the confirmatory diagnosis of UTI [20], poor access to free CDST services in the facility necessitates the use of empirical antibiotics for the management of uncomplicated UTI. Thus, the increase in empirical antibiotic prescriptions is appreciable given the low coverage of CDST [5,6]. However, it is essential to enhance access to CDST for the confirmatory diagnosis of UTI by expanding laboratory capacity to perform CDST and subsidizing CDST cost by incorporating it into the NHIS [20].

Second, the 10% relative increase in compliance with the STGs in the after cohort may be attributed to the implementation of the audit–feedback mechanism in the pharmacy and the introduction of STG orientation during medical residents’ training. These findings are similar to those from a tertiary hospital in Ghana, where antibiotic use was reduced and prescription practices improved following the introduction of education and feedback interventions as part of an antimicrobial stewardship programme [35]. Thus, to ensure the rational use of antibiotics and increase compliance with the STGs in Ghana, there is a need for implementing STG orientation and audit–feedback interventions through antimicrobial stewardship programmes established in health facilities [33].

Third, there was an increase in the use of the WHO Access category of antibiotics in the after cohort. This is in contrast to the STGs, as both antibiotics recommended for uncomplicated UTIs fall within the Watch category [20]. As some of the senior physicians refer to international guidelines (Up to Date, British National Formulary, Medscape, etc.) for the management of uncomplicated UTIs, they prefer using Access category drugs like nitrofurantoin, recommended in the guidelines. The recommendations of international guidelines align with the WHO’s push for using Access category antibiotics empirically [36]. Thus, the MoH of Ghana should consider including Access category antibiotics, such as nitrofurantoin, for the empirical management of uncomplicated UTIs in the next edition of Ghana’s STGs, which is currently being drafted.

Fourth, despite a decrease in non-compliance with the STGs while prescribing empirical antibiotics for males with uncomplicated UTI (from 93% to 82%), still, eight out of ten males were not prescribed empirical antibiotics in line with the STGs. This was mainly because only one in five males were prescribed antibiotics for 10–14 days, as recommended in the STGs. Similar low compliance with STGs was reported when longer treatment durations were recommended in other countries [15,18,37]. UTIs in men are considered severe, as they can cause long-lasting complications in the genitourinary tract; therefore, a longer duration of therapy is recommended [38]. Although recent studies have reported that five to seven days of empirical antibiotic treatment for males with uncomplicated UTI is effective [39,40], there is limited evidence from LMICs, including Ghana. Thus, there is a need for randomized controlled trials in Ghana to assess the effectiveness of shorter durations of empirical antibiotics for males with uncomplicated UTIs. Furthermore, there is an urgent need for targeted focus during training and audit–feedback activities to highlight the need for a longer duration of treatment for males with uncomplicated UTI.

In conclusion, this study showed how prescription audit through operational research using EMRs and implementation of recommendations improved the empirical treatment of uncomplicated UTIs in a primary care setting in Ghana. Given the availability of similar EMR systems in other facilities in Ghana, prescription audits through operational research should be extended to other sites and applied to various infectious diseases to improve STG compliance and promote rational antibiotic use. To achieve this, it is essential to strengthen the operational research capacity of antimicrobial stewardship programme personnel in health facilities through practical training initiatives, such as SORT IT [22]. Similar approaches in other LMICs could help curb irrational antibiotic use, reduce the burden of AMR, and save lives.

## Figures and Tables

**Table 1:** Dissemination of findings and recommendations for improving adherence to guidelines in the management of uncomplicated UTI from the operational research study conducted by Owusu et al. [23] in 2022.

Mode of Delivery	To Whom (Number)	Where	When
Three-minute lightning PowerPoint presentation	MoH stakeholders (32)	The national SORT IT module 4	October 2022
Published article	Global and national AMS/AMR professional groups (35)	Social media platforms: Whatsapp, Facebook, and LinkedIn	November 2022
Twenty-minute technical PowerPoint presentation	Polyclinic staff and other medical officers (50+)	Polyclinic morning meeting (Online)	January 2023
Ten-minute technical PowerPoint presentation	MoH stakeholders (30)	National SORT IT dissemination programme	July 2023
Poster presentation	Pharmacists from all over Ghana (500+)	Annual General Meeting of Pharmacists	September 2023
Plain language handouts	Polyclinic core management Team (5)	Polyclinic HoD office	December, 2022
	Pharmacy students and interns (100)	Polyclinic pharmacy	January 2023

Abbreviations: SORT IT—Structured Operational Research Training IniTiative; HoD—Head of Department; MoH—Ministry of Health; AMS—antimicrobial stewardship; AMR—antimicrobial resistance.

**Table 2:** List of recommendations from the operational research on compliance with the STGs for prescribing empirical antibiotics in a primary health facility by Owusu et al. [23] in 2022 and the implementation status of these recommendations as of July 2024.

Recommendation	Action Status	Details of Action (When)
Institute an audit feedback system by leveraging the EMR within a comprehensive antimicrobial stewardship programme	Fully implemented	A clinical audit–feedback system was integrated into the resident training in January 2023. Clinical audit and feedback on malaria and pneumonia management were implemented in September 2023 and March 2024, respectively. The prescribers were presented with the findings and feedback was provided on ways to improve compliance with the STGs. The antimicrobial stewardship committee with a dedicated clinical audit and feedback team is yet to be established, although efforts are far advanced.
Determine and address reasons for the poor compliance in this study	Partially implemented	This was performed informally by interviewing the residents and medical officers in February and March 2023. It was discovered that apart from the STGs, other international guidelines are used by prescribers, including the BNF, Medscape, and UpToDate.
Systematic monitoring of compliance with STGs for other diseases	Fully implemented	Monitoring of compliance with guidelines in severe malaria and pneumonia completed in September 2023 and March 2024, respectively.
Promote similar efforts to assess compliance with STGs across primary care facilities with EMRs	Partially implemented	Ongoing collaborations with 5 other Ghanaian hospitals (Police Hospital, Tema General Hospital, Keta Municipal Hospital, University of Ghana Medical Center, and Cape Coast Teaching Hospital) to determine compliance with guidelines in prescribing for community-acquired pneumonia.
Consider revising the STGs on treatment of uncomplicated UTI based on current evidence	Partially implemented	The Ministry of Health is currently reviewing the STGs

Abbreviations: EMRs—electronic medical records; HoD—Head of Department; BNF—British National Formulary; STGs—standard treatment guidelines.

**Table 3:** Comparison of socio-demographic, clinical, and prescriber characteristics of adults with uncomplicated UTI diagnosed in the outpatient department of the Korle Bu Polyclinic/Family Medicine Department of Ghana before (October 2019 to October 2021) and after (January 2024 to December 2024) the operational research conducted in 2022.

Characteristics	Before		After		*p* Value ^4^
	n	(%) 1	n	(%) 1	
**Total**	3717	(100)	3457		
**Age in years**					
18–29	757	(20.4)	730	(21.1)	0.130
30–44	919	(24.7)	775	(22.4)	
45–59	804	(21.6)	787	(22.8)	
≥60	1237	(33.3)	1165	(33.7)	
**Gender**					
Male	1064	(28.6)	974	(28.2)	0.672
Female	2653	(71.4)	2483	(71.8)	
**NHIS**					
Yes	2920	(78.5)	2718	(78.6)	0.473
No	797	(21.5)	739	(21.4)	
**Comorbidities ^2^**					
Diabetes mellitus	621	(16.7)	644	(18.6)	0.016
Hypertension	1231	(33.1)	1327	(38.4)	<0.001
**Routine urine examination**					
Not done	1137	(30.5)	1138	(32.9)	0.007
Done	2574	(69.3)	2319	(67.1)	
Missing	6	(0.2)			
**Prescriber sex**					
Male	2084	(56.0)	1783	(51.6)	<0.001
Female	1623	(43.7)	1674	(48.4)	
Missing	10	(0.3)			
**Prescriber rank ^3^**					
Physician Assistant	36	(1.0)	32	(1.0)	<0.001
Medical Officer	1616	(43.5)	1466	(42.4)	
Senior/Principal/Deputy Chief/Chief Medical Officer	348	(9.4)	554	(16.0)	
Resident/Senior Resident	1136	(30.6)	1103	(31.9)	
Specialist/Senior Specialist/Consultant	469	(15.6)	302	(8.7)	

^1^ Column percentage. ^2^ Multiple comorbidities are possible. ^3^ Those joining the service immediately after graduation are designated as medical officers. Based on their years of service, the medical officers progress through the ranks of the senior medical officer, deputy chief medical officer, and chief medical officer. Doctors with postgraduate specialization join as senior residents and progress through the ranks of specialist, senior specialist, and consultant. ^4^ Chi-square test. UTI—urinary tract infection; NHIS = National Health Insurance Scheme.

**Table 4:** Compliance with STGs for management of adult patients with uncomplicated UTI diagnosed at the Korle Bu Polyclinic/Family Medicine Department of Ghana before (October 2019 to October 2021) and after (January 2024 to December 2024) the operational research conducted in 2022.

Particulars	Before			After			*p* Value ^2^
	N	n	(%) 1	N	n	(%) 1	
Prescribed any empirical antibiotic	3717	3073	(83)	3457	2986	(86)	<0.001
Prescribed the empirical antibiotic recommended in the STGs ^3^	3073	2714	(88)	2986	2719	(91)	<0.001
Prescribed the empirical antibiotic recommended in the STGs at the correct dose ^4^	2714	2712	(~100)	2719	2717	(~100)	0.996
Prescribed the empirical antibiotic recommended in the STGs for the correct duration ^4^	2714	1848	(68)	2719	1977	(73)	<0.001
Prescribed the empirical antibiotic recommended in the STGs in the correct route ^4^	2714	2712	(~100)	2719	2719	(100)	0.156
Prescribed the empirical antibiotic recommended in the STGs in the correct frequency ^4^	2714	2712	(~100)	2719	2718	(~100)	0.561
Prescribed the empirical antibiotic in line with the STGs ^5^	3073	1847	(60)	2986	1975	(66)	<0.001

^1^ Percentage calculated with preceding N as the denominator. ^2^ *p* value calculated using chi-squared test. ^3^ Prescription of either oral ciprofloxacin or cefuroxime as recommended in the STGs among those who were prescribed empirical antibiotics. ^4^ Dose, duration, and frequency in line with the STGs when either oral ciprofloxacin or cefuroxime was prescribed. ^5^ Prescription of either oral ciprofloxacin or cefuroxime in the dose, frequency, and duration recommended in the STGs among those who received empirical antibiotics.

**Table 5:** Distribution of empirical antimicrobials across WHO AWaRe categories prescribed for adults diagnosed with uncomplicated UTI in the outpatient department of the Korle Bu Polyclinic/Family Medicine Department of Ghana before (October 2019 to October 2021) and after (January 2024 to December 2024) the operational research conducted in 2022.

Antimicrobials	Before, N = 3378		After, N = 3491		*p* Value ^b^
	n	(%) ^a^	n	(%) ^a^	
***Access***					
**Total**	**381**	**(11.2)**	**577**	**(16.5)**	**<0.001**
Tinidazole	119	(3.5)	121	(3.5)	0.898
Nitrofurantoin	90	(2.7)	142	(4.1)	<0.001
Doxycycline	88	(2.6)	157	(4.5)	<0.001
Amoxicillin/clavulanic acid	52	(1.5)	22	(0.6)	<0.001
Secnidazole	20	(0.6)	38	(1.1)	0.025
Metronidazole	8	(0.2)	72	(2.0)	<0.001
Clindamycin	2	(0.1)	13	(0.4)	0.005
Amoxicillin	1	(<0.1)	7	(0.2)	0.038
Sulfamethoxazole/trimethoprim	1	(<0.1)	5	(0.1)	0.111
***Watch***					
**Total**	**2997**	**(88.8)**	**2914**	**(83.5)**	**<0.001**
Cefuroxime	1831	(54.2)	1479	(42.4)	<0.001
Ciprofloxacin	1036	(30.7)	1252	(35.9)	<0.001
Cefixime	38	(1.1)	86	(2.5)	<0.001
Ceftriaxone	33	(1.0)	17	(0.5)	0.017
Levofloxacin	31	(0.9)	18	(0.5)	0.048
Azithromycin	23	(0.7)	50	(1.4)	0.002
Cefpodoxime	3	(0.1)	3	(0.1)	0.968
Clarithromycin	2	(0.1)	9	(0.2)	0.040

^a^ Column percentage with total number of antibiotics as the denominator. ^b^ Chi-squared test. UTI = urinary tract infection; AWaRe = Access, Reserve, and Watch; WHO = World Health Organization.

**Table 6:** Patient and prescriber characteristics associated with the prescription of empirical antibiotics not in line with the STGs among adults prescribed any empirical antibiotic for uncomplicated UTI in the outpatient department of the Korle Bu Polyclinic/Family Medicine Department of Ghana after (January 2024 to December 2024) the operational research conducted in 2022.

Characteristics	Total	Empirical Antibiotics not in Line with STGs		Unadjusted ^b^		Adjusted ^c^	
		n	(%) ^a^	PR	(95% CI)	aPR	(95% CI)
**Total**	2986	1011	(33.9)				
**Age in years**							
18–29	628	168	(26.8)	1		1	
30–44	660	216	(32.7)	1.2	(1.0–1.4)	1.1	(0.9–1.3)
45–59	685	252	(36.8)	1.4	(1.2–1.6)	1.1	(0.9–1.3)
≥60	1013	375	(37.0)	1.4	(1.2–1.6)	1.1	(0.9–1.3)
**Gender**							
Male	844	689	(81.6)	**5.4**	**(4.9–6.0)**	**5.4**	**(4.9–6.1)**
Female	2142	322	(15.0)	1		1	
**NHIS**							
Yes	2568	851	(33.1)	1		1	
No	418	160	(38.3)	1.2	(1.0–1.3)	1.0	(0.8–1.1)
**Comorbidities ^d^**							
Diabetes mellitus	537	181	(33.7)	1.0	(0.9–1.1)	1.1	(1.0–1.2)
Hypertension	1142	419	(36.7)	1.1	(1.0–1.3)	1.0	(0.9–1.1)
**Routine urine examination**							
Done	960	333	(34.7)	1.0	(0.9–1.2)	1.0	(1.0–1.1)
Not done	2026	678	(33.5)	1		1	
**Prescriber gender**							
Male	1537	523	(34.0)	1.0	(0.9–1.1)	1.0	
Female	1449	488	(33.7)	1		1	
**Prescriber rank ^e^**							
Physician Assistant	25	10	(40.0)	1.2	(0.8–2.0)	1.3	(0.8–2.1)
Medical Officer	1269	439	(34.6)	1.1	(0.9–1.2)	1.0	(0.9–1.1)
Senior/Principal/Deputy Chief/Chief Medical Officer	484	167	(34.5)	1.1	(0.9–1.2)	1.0	(0.9–1.1)
Resident/Senior Resident	957	310	(32.4)	1		1	
Specialist/Snr Specialist/Consultant	251	85	(33.9)	1.0	(0.9–1.3)	1.0	(0.9–1.2)

^a^ Row percentage; ^b^ log binomial regression; ^c^ modified Poisson regression with all the patient and prescriber characteristics included and categories in bold are statistically significant; ^d^ not having the specific comorbidity is the reference; ^e^ Those joining the service immediately after graduation are designated as medical officers. Based on their years of service, the medical officers progress through the ranks of the senior medical officer, principal medical officer, deputy chief medical officer, and chief medical officer. Doctors with postgraduate specialization join as residents and progress through the ranks of senior residents, specialist, senior specialist, and consultant. UTI = urinary tract infection; STGs = standard treatment guidelines; PR = prevalence ratio; CI = confidence interval; aPR = adjusted prevalence ratio.

## Data Availability

The raw data supporting the conclusions of this article will be made available by the authors on request.
